# Echo-endoscopic drainage of retrogastric pancreatic pseudocysts as a bridge-to-surgery for complicated cases of duodenal duplication cyst: case report

**DOI:** 10.3389/fped.2025.1588823

**Published:** 2025-06-26

**Authors:** Joseph Xavier Pacifique, Nicolas Jauquier, Natalie Divjak, Sebastien Godat, Sabine Vasseur Maurer

**Affiliations:** ^1^ Department of Surgery, Etablissement Hospitalier du Nord Vaudois eHnv, Yverdon-les-Bains, Switzerland; ^2^ Department of Pediatric Surgery, Centre Hospitalier Universitaire Vaudois CHUV, Lausanne, Switzerland; ^3^ Department of Gastro-enterology, Centre Hospitalier Universitaire Vaudois CHUV, Lausanne, Switzerland

**Keywords:** duodenal duplication cyst, echo-endoscopy, pancreatic pseudocyst, cystogastric stent, bridge to surgery, case report

## Abstract

**Introduction:**

Duodenal duplication cysts (DDC) are rare congenital malformations which are generally diagnosed in the first decade of life. The clinical presentation of DDC is highly variable and may be complicated by pancreatitis. When pancreatic pseudocysts (PPC) develop, definitive DDC treatment is delayed and exposes the patient to recurrent episodes of pancreatitis which further lengthen the process. We present a novel approach to the management of such cases by using echo-endoscopic cystogastric drainage of a large retrogastric PPC as a bridge to surgery. To our knowledge, this is the youngest reported case.

**Case:**

A 21-month-old girl presented with abdominal pain, bloating, vomiting and failure to thrive lasting for 3 months. Her prior medical history was normal.

**Diagnosis, therapeutic intervention and outcomes:**

Blood work showed pancreatitis. Ultrasound (US) showed multiple cysts inside the abdomen. A thoraco-abdominal magnetic resonance imaging (MRI) scan allowed differentiation between multiple PPC and a DDC, which had caused a complicated obstructive pancreatitis. The DDC was confirmed by biopsies. Further imaging identified a large persistent retrogastric pseudocyst. Due to poor feeding and stable but compromised general condition, a two-step procedure was scheduled with echo-endoscopic cystogastric drainage of the large retrogastric PPC to reduce the convalescence time after the last episode of pancreatitis, followed by surgical resection of the DDC. The patient was released from the hospital the day after this procedure as oral intake had normalized. Unfortunately, 3 weeks after this procedure, the patient developed a septic shock due to infection of the remaining cysts. As surgery was required to treat the sepsis, the DDC was resected at the same time.

**Conclusion:**

Echo-endoscopic cystogastric drainage is feasible and effective in children as young as 21 months. Pediatric guidelines have yet to be determined for this procedure.

## 1 Introduction

Digestive tract duplications are congenital malformations situated on the mesenteric side of the digestive tract, which share a common blood supply with the native bowel. They can occur anywhere from the mouth to the anus. 85% of them are diagnosed before the age of 2, with 60% before 6 months (1). Duodenal duplication cysts (DDC) represent 2%–12% of digestive tract duplications and have an estimated prevalence of 1/100,000 live births (2). Most DDCs occur in continuity with the second and third parts of the duodenum, sharing a common muscularis propria (3). Some occur as separate entities within or around the pancreas, between the inferior vena cava and portal vein, or adherent to the stomach (4, 5). Pancreatitis is a well-documented complication of DDCs. When pancreatic pseudocysts (PPC) complicate an episode of pancreatitis caused by a DDC, a vicious cycle can ensue where the surgical treatment of the DDC is delayed because of recurring episodes of pancreatitis. Challenges in these cases range from diagnosis to treatment.

This case is presented to share our experience with echo-endoscopic drainage of retrogastric PPC as a bridge to surgery in complicated cases of pancreatitis resulting from DDC. To our knowledge, this is the youngest case where this procedure has been used. This approach is uncommon in children, due to equipment size limitations, but is slowly gaining in popularity due to industry improvements (6).

## 2 Case report

### 2.1 Timeline of the main events

**Figure 1:**
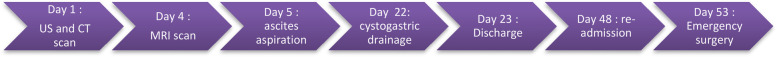
Timeline of main events.

**Day 1:** We present the case of a 21-month-old girl who was brought to the emergency department of a regional hospital in Switzerland for acute abdominal pain in October of 2022 (Figure 1). Her previous medical history was normal, including during pregnancy. For four months, the patient had been suffering from isolated episodes of abdominal pain, distension and low-grade fever which were attributed to viral infections. The reason for the consultation in the emergency department was the refusal of solid food intake for 4 days which resulted in the loss of 400 g. The recent medical history showed no gastro-intestinal, respiratory, ENT or other systemic symptoms which could orient the diagnosis. There was also no history of abdominal or other trauma. The vital signs were as follows: HR 156/min, RR 32/min, Temperature 36.2°C, O2 saturation 97%, a weight of 10.2 kgs and normal hydration and perfusion status. The abdomen was distended, the auscultation was normal, and an uncomplicated umbilical hernia was present. Complete physical examination was otherwise normal. The initial blood work showed a normal white blood cell count (WBC), normal hemoglobin, and a C-Reactive Protein level (CRP) of 30.5 mg/L.

**Figure 2:**
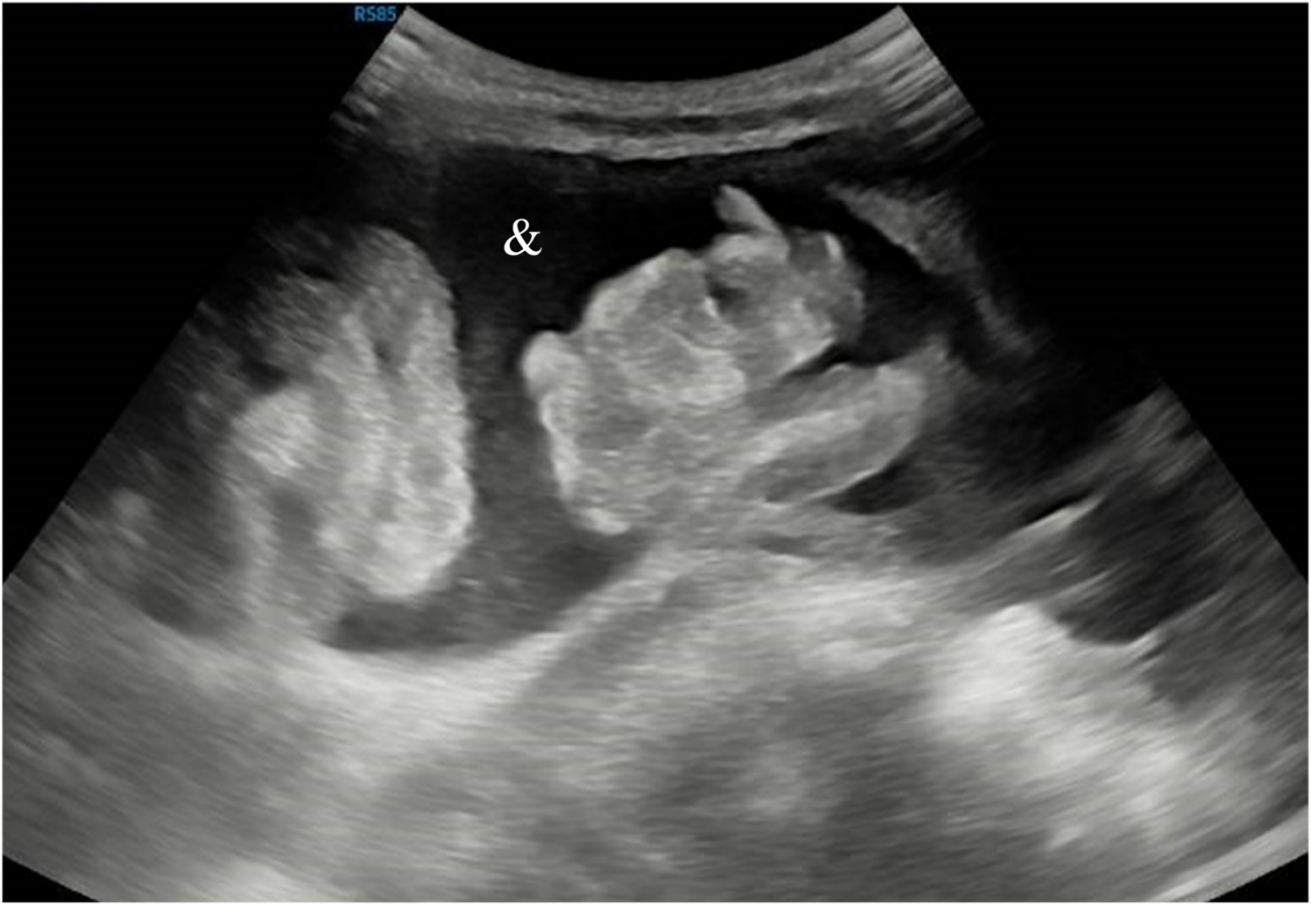
Abdominal US showing free fluid (&).

An abdominal ultrasound (US) (Figure 2) was performed which showed multiples cysts and ascites.

**Figures 3 and 4:**
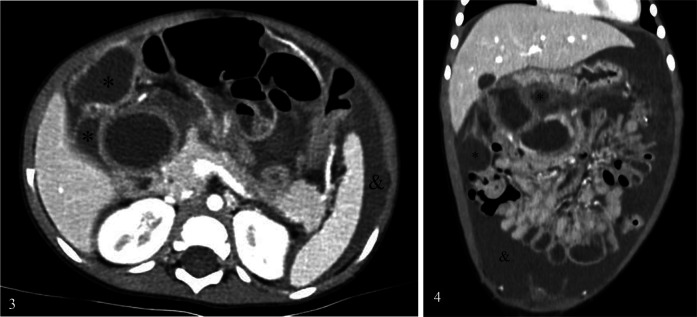
CT scan showing multiple cysts (*) and free fluid (&).

This was followed by a thoraco-abdominal computed tomography (CT) scan (Figures 3, 4) which confirmed the presence of multiple, large, abdominal cysts and free abdominal fluid. As cystic lymphangioma was suspected, the patient was transferred to a tertiary hospital center for etiologic diagnosis and treatment. Extensive blood work showed Lipase 667 UI/L, CRP 30 mg/L, normal liver function tests, and normal alpha-feto-protein and Beta-hCG levels. The criteria for pancreatitis were met and the patient was treated accordingly.

**Figures 5 and 6:**
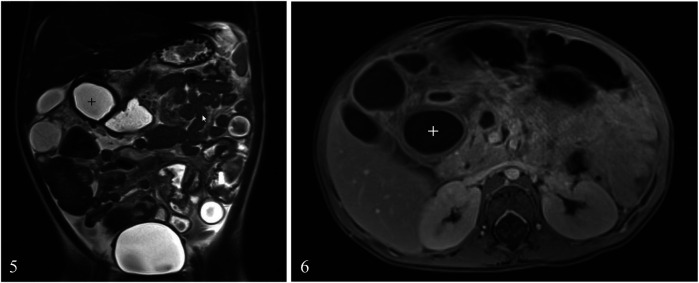
MRI scan showing double-walled cyst (+).

**Day 4**: A thoraco-abdominal magnetic resonance imaging (MRI) scan (Figures 5, 6) was performed which showed multiple thin-walled cysts and a single thick-walled paraduodenal cyst compatible with a DDC. A naso-jejunal feeding tube (NJFT) was placed and standard enteral nutrition (Nutrini®) was administered.

**Day 5**: As the initial differential diagnosis included cystic lymphangioma, an US-guided ascites and cyst aspiration was also performed. The culture was sterile and no malignant cells were present. The analysis of the fluid showed a normal triglyceride and WBC count, but lipase and pancreatic amylase were 1,159 UI/L and 863 UI/L respectively, suggesting that the thin-walled cysts were PPC. The double-walled cyst was then highly suspected to be a DDC. The patient then presented intercurrent rotavirus gastroenteritis delaying further investigations.

**Figure 7:**
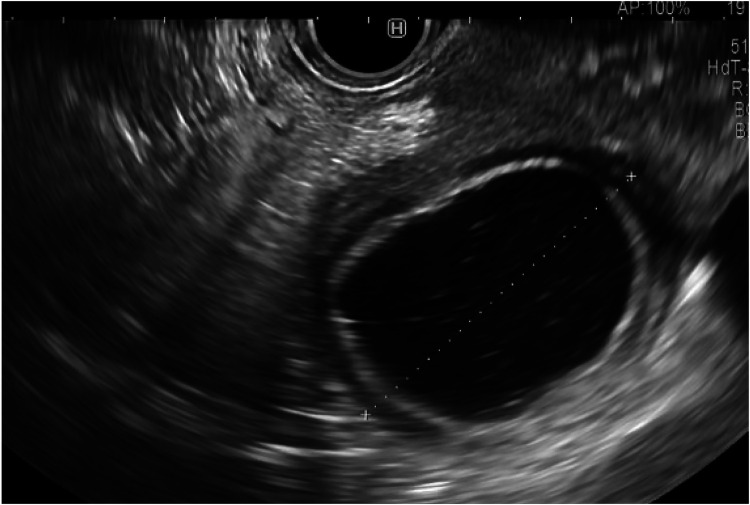
Endo-US showing double-walled cyst.

**Figure 8:**
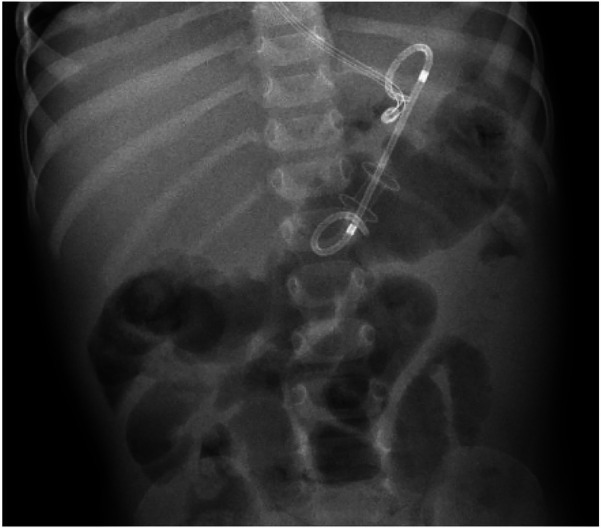
X-ray showing stent and pigtail.

**Day 22**: Two weeks after admissions, as investigations into the etiologic diagnosis started again, the patient showed a stagnant weight curve, poor oral intake due to gastroparesis and low jejunal feeding tolerance. Along with biopsies of the suspected DDC, a drainage of the retrogastric PPC was decided to alleviate gastroparesis symptoms and speed-up recovery. The echo-endoscopy confirmed the presence of a single thick-walled DDC, signs of inflammation involving the pancreatic head and several thin-walled PPC, the largest being retrogastric. A Cystogastric drainage was performed using the smallest stent available, a Hot Axios 6 mm stent. A Pigtail 7Fr was placed through it (Figures 7, 8). The drainage revealed a white fluid which was positive for anaerobes. Biopsy of the suspected duplication cyst showed digestive epithelial lining confirming that the cyst was a DDC.

**Day 23**: Normal oral intake and enteral nutrition were well tolerated after this procedure. The patient was released from the hospital the day after the procedure with painkillers and ambulatory nutritional support via our dietician team. The removal of the stent was scheduled 6 weeks after its placement.

**Day 30**: At the 1-week follow-up consultation, the patient kept a stable weight but suffered from intermittent abdominal pain only during feeding. The dietary prescription was changed, and the patient went back home.

**Day 48**: The patient was hospitalized for abdominal pain and fever. The WBC was 9 G/L and CRP was 280 mg/L. A Norovirus was found in the stool test. The MRI scan showed no residual retrogastric PPC, but multiple infected PPC. A large spectrum antibiotic therapy was initiated.

**Day 53**: The patient developed a septic shock and presented increased signs of infection on the control MRI scan. A laparotomy was performed which found multiple infected pseudo-cysts and a 6 cm long, perforated, tubular, non-communicating, duodenal duplication, on the mesenteric side of D1. The dome of the cyst and the mucosa found on the common wall were excised and sent for analysis. The gastro-cystic stent, which had spontaneously migrated in the omental bursa, was removed and the wall of the stomach was closed. Lavage and drainage of the infected collections was performed before closure. The pathology report showed a DDC with typical duodenal wall characteristics. The antibiotics were stopped 10 days after surgery and the patient was released from the hospital on day 78.

**Figures 9 and 10:**
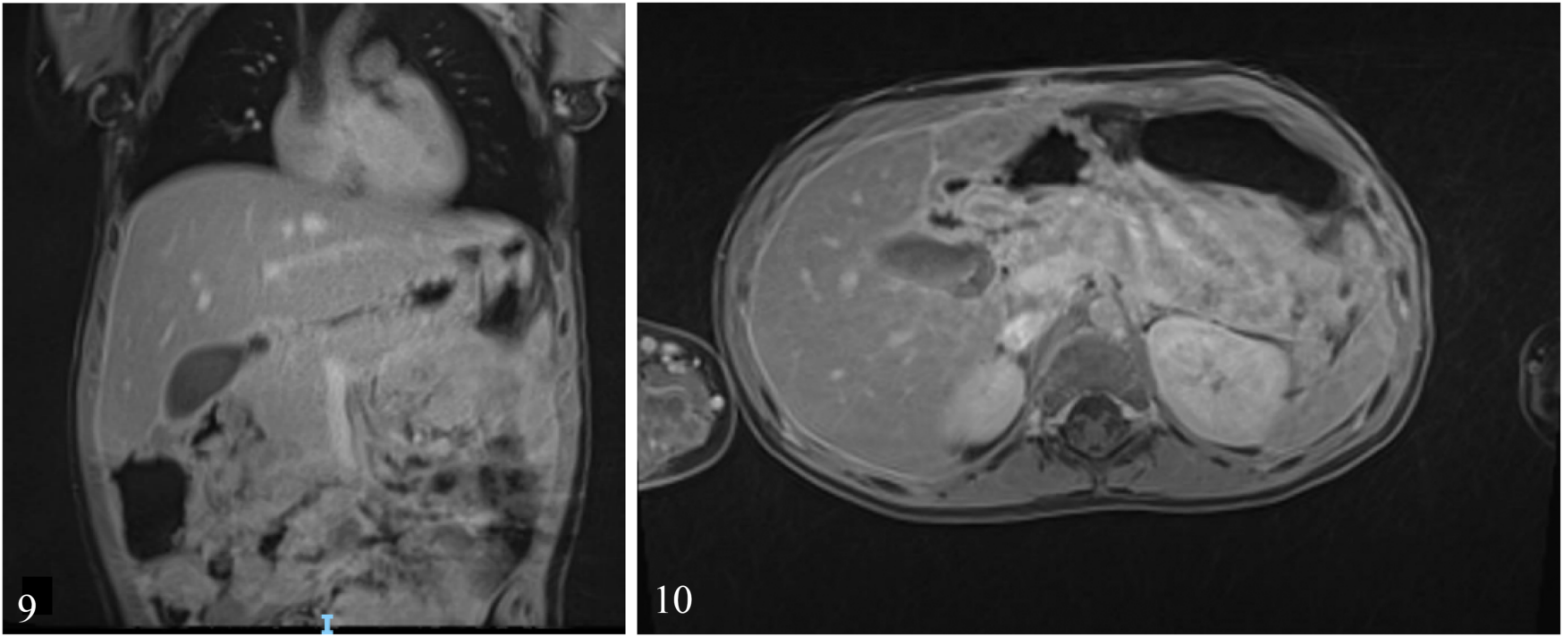
MRI scan showing no residual DDC or PPC 3 months after surgery.

**Day 151**: Three months after surgery, a control echo-endoscopy was performed which showed no residual DDC. An MRI scan was also performed at the same time which also showed no residual DDC and no residual collections or pseudocysts (Figures 9, 10). At the 1-year follow-up consultation, the patient had caught-up on her growth curves and was thriving. The 1-year control US was normal.

Unfortunately, 15 months after surgery, the patient presented with adhesion-related small bowel obstruction which did not respond to conservative treatment. She had an exploratory laparotomy with extensive adhesiolysis and a short segmental small bowel resection. Clinical course was thereafter uneventful.

## 3 Discussion

Pancreatitis has an incidence of 1/10,000 children per year, and this rate is increasing (7). Anatomical variations should be suspected and investigated in children with recurrent acute pancreatitis. DDC are such anatomical variations and cause nonspecific symptoms such as abdominal pain, nausea and vomiting. Common complications include volvulus, intussusception, recurrent hemorrhage and perforation (8). Pancreatitis is the most reported complication of DDCs and is caused by 3 main mechanisms: (1) transient obstruction of the major papilla outflow by the nearby cyst due to peristalsis; (2) compression of the main pancreatic duct by a large cyst; and (3) obstruction of the pancreatic duct by biliary sludge, viscous mucous secretions or blood clots from the cyst (9). We suspect that the second mechanism caused the symptoms of our patient.

In our case, establishing the diagnosis was challenging. The CT scan showed only undifferentiable cysts. Although the MRI scan suggested a DDC, the definitive diagnosis was confirmed after the pathology results were obtained. Only then was surgical resection of the DDC planned. Regarding the imagery modalities used, we argue that for complex cases such as this, MRI scans, rather that CT scans, should be favored for the following reasons: (1) 5%–20% of children with pancreatitis have pancreato-biliary anomalies, for which MRI scans are the imaging gold standard (10); (2) MRI scans are equally effective as CT scans for severity scoring and follow-up of pancreatitis in children (11); (3) MRI scans provide detailed anatomical images of the DDC and its blood supply, facilitating surgical planning.

Surgical, endoscopic, and percutaneous methods have been described for the drainage of PPCs in pediatric patients. No specific guidelines compare these approaches in this population. Generally, adult guidelines are adapted for pediatric use. The 2019 European Society of Gastrointestinal Endoscopy (ESGE) guidelines recommend echo-endoscopic drainage of accessible PPCs in chronic pancreatitis, over percutaneous or surgical treatment (12). The American Gastroenterological Association (AGA) has not published specific guidelines for PPCs. However, their 2020 Clinical Practice Update on the management of pancreatic necrosis (13) aligns with the 2019 ESGE guidelines. It recommends echo-endoscopic drainage as the preferred initial method for PPCs, percutaneous drainage when endoscopic drainage is unavailable, unsuccessful or not feasible due to the risk of pancreaticocutaneous fistulae, and surgery as last resort for infected pancreatic necrosis or sterile pancreatic necrosis causing persistent organ dysfunction or failure to thrive.

Although echo-endoscopic drainage of retrogastric PPCs is commonly performed in adults, these procedures have recently been conducted in pediatric patients with good outcomes. A PubMed literature review of PPC managed with endoscopic drainage, using search terms including “PPC”, “walled-off necrosis”, “endoscopic ultrasound”, “cystogastric stent” and “pediatric”, yielded 211 results. Of these, 41 studies were relevant, primarily case reports and retrospective case series. The only meta-analysis, by Nabi et al., summarized the best case series (14). The youngest patient in these studies was 2 years old (15). Table 1 gives a brief summary of some of these studies.

**Table 1:** Characteristics and outcomes of studies on Echo-endoscopic drainage of pancreatic fluid collections in children.

Study	Country	Age (years)	N	Size of PFC (cm)	Etiology	Stent	Adverse events	Technical success (%)	Clinical success	Re-intervention	Follow-up (months)
Nabi et al. 2017 (16)	India	13 ± 3.4	30	6.1–17.5	Trauma 6 Biliary 1 Idiopathic 23	Plastic	10 (2 major bleeding, perforation)	29 (96.7)	28 (93.3)	3	5–41 mo
Ramesh et al. 2013 (17)	USA	8.4 ± 2.1	7	12.3 ± 2.6	Trauma 5 Idiopathic 1 Heredity 1	Plastic	0	7 (100)	5 (71.4)	2	34 mo
Bang et al. 2016 (18)	USA	13.5 ± 3.1	6	13.3 ± 6.3	Idiopathic 3 Biliary 2 Drug 1	Plastic 5 Metal 1	0	6 (100)	4 (66.7)	2	29 mo
Nabi et al. 2019 (19)	India	9–18	32	NR	Biliary 2 Idiopathic 26 Alcohol 2 Divisum 1 Eosinophilic 1	Metal	NR	32 (100)	29 (90.6)	3	15 mo
Farr et al. 2020 (20)	USA	NR	5	10.6 ± 3.4	Trauma 5	Plastic 3 Metal 2	NR	5 (100)	5 (100)	NR	23 mo
Lal et al. 2020 (21)	India	10–11	6	7.6–14.7	NR	Plastic 1 Metal 5	0	6 (100)	6 (100)	NR	NR

PFC, pancreatic fluid collection; NR, not reported; mo, months.

Complications of echo-endoscopic drainage of PPCs include bleeding, hematemesis, intestinal perforation, infection, and stent migration (16). External stent migration is generally benign, whereas internal migration, as in our case, requires endoscopic or surgical intervention. Double-pigtail stents are recommended to reduce migration. Metal stents are associated with higher bleeding risk. To reduce perforation risk, echo-endoscopy and fluoroscopy can be used (14).

In our case, a conservative approach to the retrogastric PPC was not feasible because the patient suffered from severe gastroparesis. Oral intake and NJFT were significantly reduced, slowing recovery and exposing the patient to recurrent episodes of pancreatitis. Surgery was not considered as a viable option because intra-abdominal inflammation due to pancreatitis increased the risk of complications, such as bleeding and bowel perforation. Percutaneous drainage was deemed unsuitable for our case as echo-endoscopy was readily available. Thus, echo-endoscopic drainage of the retrogastric PPC was planned as a bridge-to-surgery. The procedure was successful, markedly improving tolerance of oral intake and NJFT and significantly reducing abdominal discomfort. The patient was discharged the day after the procedure, while waiting for surgery.

We do not believe that the biopsy of the DDC or the ascites aspiration caused the septic shock, as they were performed 3 and 4 weeks prior, respectively. Additionally, the DDC perforation is unlikely to have caused the septic shock because the cyst was non-communicating. Instead, we hypothesize that the cystogastric stenting infected all the PPCs, as they were initially connected. Consequently, the septic shock may have resulted from one of two mechanisms. First, the migration of the stent impaired drainage. Alternately, as the PPC decreased in size over time, some became isolated and were no longer drained.

The preferred treatment for DDC is complete surgical resection when feasible to prevent malignant transformation (22). Partial resection or internal derivation is recommended if bilio-pancreatic structures are connected to the DDC (23). Endoscopic marsupialization, an emerging technique, has been described for partial cyst resection (24). In our case, due to the septic shock, surgery was performed.

Written informed consent was obtained from the patient's legal guardians for the publication of this case. When asked about their perspective, the guardians reported feeling relieved after months of uncertainty once the diagnosis and treatment plan were established. They also reported feeling heard by the medical and care teams. Their questions were answered in clear, jargon-free language, and they actively participated in the decision-making process. They also expressed frustration with initial pain management due to their daughter's high pain tolerance. They believed treatment delays could have been avoided with direct access to senior surgeons, the primary decision-makers, rather than consulting interns first, particularly on weekends.

## 4 Conclusion

The diagnosis and management of acute recurrent pancreatitis complicated by multiple PPCs caused by a DDC can be challenging. Cystogastric drainage of retrogastric PPCs is an effective and feasible bridge-to-surgery in children as young as 21 months old. It can speed up recovery and enable earlier surgical intervention for the DDC.

Due to the rarity of echo-endoscopic drainage of retrogastric PPCs in children, specific guidelines for this procedure are lacking. Rather, adult guidelines are adapted for pediatric cases. The increasing incidence of pediatric pancreatitis and the availability of pediatric echo-endoscopes and stents highlight the need for prospective studies. These should determine optimal timing, stent types, and PPC sizes requiring drainage.

## Data Availability

The original contributions presented in the study are included in the article, further inquiries can be directed to the corresponding author.
